# Evaluation of US Hospital Episode Spending for Acute Inpatient Conditions After the Patient Protection and Affordable Care Act

**DOI:** 10.1001/jamanetworkopen.2020.23926

**Published:** 2020-11-23

**Authors:** Andrew M. Ibrahim, Ushapoorna Nuliyalu, Emily J. Lawton, Stephen O’Neil, Justin B. Dimick, Baris Gulseren, Shashank S. Sinha, John M. Hollingsworth, Tedi A. Engler, Andrew M. Ryan

**Affiliations:** 1 Institute for Healthcare Policy and Innovation, University of Michigan, Ann Arbor; 2 School of Public Health, University of Michigan, Ann Arbor; 3 National University of Ireland Galway, Galway, Ireland

## Abstract

**Importance:**

Under the Patient Protection and Affordable Care Act (ACA), US hospitals were exposed to a number of reforms intended to reduce spending, many of which, beginning in 2012, targeted acute care hospitals and often focused on specific diagnoses (eg, acute myocardial infarction, heart failure, and pneumonia) for Medicare patients. Other provisions enacted in the ACA and under budget sequestration (beginning in 2013) mandated Medicare fee cuts.

**Objective:**

To evaluate the association between the enactment of ACA reforms and 30-day price-standardized hospital episode spending.

**Design, Setting, and Participants:**

This policy evaluation included index discharges between January 1, 2008, and August 31, 2015, from a national random 20% sample of Medicare beneficiaries. Data analysis was performed from February 1, 2019 to July 8, 2020.

**Exposure:**

Payment reforms after passage of the ACA.

**Main Outcomes and Measures:**

30-day price-standardized episode payments. Three alternative estimation approaches were used to evaluate the association between reforms following the ACA and episode spending: (1) a difference-in-difference (DID) analysis among acute care hospitals, comparing spending for diagnoses commonly targeted by ACA programs with nontargeted diagnoses; (2) a DID analysis comparing acute care hospitals and critical access hospitals (not exposed to reforms); and (3) a generalized synthetic control analysis, comparing acute care and critical access hospitals. Supplemental analysis examined the degree to which Medicare fee cuts contributed to spending reductions.

**Results:**

A total of 7 634 242 index discharges (4 525 630 [59.2%] female patients; mean [SD] age, 79.31 [8.02] years) were included. All 3 approaches found that reforms following the ACA were associated with a significant reduction in episode spending. The DID estimate comparing targeted and untargeted diagnoses suggested that reforms following the ACA were associated with a −$431 (95% CI, −$492 to −$369; −2.87%) change in total spending, while the generalized synthetic control analysis suggested that reforms were associated with a −$1232 (95% CI, −$1488 to −$965; −10.12%) change in total episode spending, amounting in a total annual savings of $5.68 billion. Cuts to Medicare fees accounted for most of these savings.

**Conclusions and Relevance:**

In this policy evaluation, the ACA was associated with large reductions in US hospital episode spending.

## Key Points

**Question:**

Were policy reforms enacted as part of the US Patient Protection and Affordable Care Act (ACA) and budget sequestration associated with reductions in hospital episode spending for acute inpatient hospitalizations?

**Findings:**

In this policy evaluation that included 7 634 242 episodes from Medicare beneficiaries, alternative estimates indicated that reforms following the ACA were associated with changes in spending between −$431 and −$1232 per episode (approximately −3% to −10%). Cuts to Medicare reimbursement accounted for most of this reduction.

**Meaning:**

These findings suggest that reforms following the ACA were associated with large reductions in Medicare spending.

## Teaser

This economic evaluation estimates the association between the enactment of Patient Protection and Affordable Care Act (ACA) reforms and 30-day price-standardized hospital episode spending.

## Introduction

The US Patient Protection and Affordable Care Act (ACA)—signed into law in March 2010—included numerous provisions to improve hospital quality and reduce Medicare spending for acute episodes. Many of these reforms, including Hospital Value-Based Purchasing,^1^ the Hospital Readmissions Reduction Program (HRRP),^2^ and the Bundled Payment for Care Improvement,^3^ disproportionately targeted 3 medical diagnoses: acute myocardial infarction, heart failure, and pneumonia.^4^ Other provisions, such as mandated payment cuts, applied more broadly across hospital care.^5,6^ In 2013, additional cuts to Medicare fees through the budget sequestration process took effect.^7^

The association between the ACA, budget sequestration, and Medicare spending for acute episodes is unclear. Increases in care coordination and efficiency during the episode, potentially spurred by payment reforms like the HRRP, the Medicare Shared Savings Program, and bundled payment programs, may have reduced hospital spending.^2,8,9,10^ At the same time, the effects of the HRRP may be overstated through high comorbidity coding^11^ and secular changes in admission rates^12^; the effectiveness of bundled payment outside of joint replacement appears to be limited^13^; and the Medicare Shared Savings Program has not effectively engaged hospitals^14^ and may be biased by selective attrition.^10^ Medicare fee cuts following these reforms reduced spending for specific services but may be offset by the use of additional services elsewhere in the episode.^15,16,17^ As a result, the net association of reforms following the ACA with hospital episode spending is uncertain.

In this context, we used national Medicare data to evaluate the association between reforms following the passage of the ACA and hospital episode spending. We used 3 alternative estimation strategies to test for changes in hospital episode spending after the initiation of the ACA.

## Methods

### Data Sources

We used US national Medicare claims from a random 20% sample of Medicare beneficiaries, including data from the Carrier, Outpatient, and Medicare Provider Analysis and Review research identifiable files. We included claims from fee-for-service beneficiaries discharged between January 1, 2008, and August 31, 2015. Additionally, we merged these data with the Annual Survey of the American Hospital Association^18^ and the Centers for Medicare & Medicaid Services (CMS) public use file on geographic variation^19^ to obtain additional hospital characteristics. This study was approved by the University of Michigan institutional review board and deemed exempt owing to the use of secondary data. This study followed the relevant sections of the Strengthening the Reporting of Observational Studies in Epidemiology (STROBE) reporting guideline.

### Study Cohort

Our study cohort included Medicare fee-for-service beneficiaries aged 66 years or older who were enrolled in both Medicare Part A and Part B and had discharges between January 1, 2008, and August 31, 2015. We defined index hospitalizations and readmissions using the specifications similar to the CMS 30-day readmission measures. Specifically, a patient’s first admission during the study period is an *index admission*. Any admission that occurs within 30 days of the index admission is a *readmission*. Consequently, admissions were classified as either an index admission or a readmission but not both. Patients who were transferred to another hospital, died during the episode, were discharged against medical advice, enrolled in Medicare Advantage, were enrolled in both Medicare and Medicaid (because of potential omissions in episode spending due to payment from Medicaid), did not have at least 12 months of claims data prior to their admission (necessary for severity adjustment), or did not have 30 days of postdischarge data available were excluded.

Targeted medical diagnoses (ie, acute myocardial infarction, heart failure, and pneumonia) were identified using *International Classification of Diseases, Ninth Revision, Clinical Modification* (*ICD-9-CM*) codes that were applied in prior studies.^2,20^ Discharges after August 31, 2015, were excluded to avoid changes associated with the retirement of the *ICD-9* and the implementation of *International Statistical Classification of Diseases and Related Health Problems, Tenth Revision *(*ICD-10*) (eFigure 1 in the Supplement).

### Measuring Episode Spending

Our primary outcome was price-standardized 30-day total episode spending. This was defined as the sum of payments related to the index hospitalization (diagnosis-related group [DRG] payments and outlier payments), physician services (inpatient and outpatient), readmissions, hospital outpatient care, and postacute care from the date of discharge through 30 days following discharge. We also examined each of these spending components as a separate outcome.

All payments were price-standardized by removing adjustments to payments that were associated with Indirect Medical Education, the Disproportionate Share Index, and with geography-based cost of living and wage indices. Price standardization was performed using methods originally described by the Medicare Payment Advisory Commission and subsequently by the Dartmouth Institute*.*^21,22,23^ This approach has been applied in several prior studies assessing Medicare payments.^24,25^ To account for inflation across the 7 years of payment data included in this study, all amounts were adjusted to 2015 US dollars.

### Statistical Analysis

Our approach to estimating the association between reforms following the ACA and episode spending was motivated by an attempt to identify the most appropriate counterfactual outcome. In other words, we sought to identify what hospital spending would have been had the ACA and subsequent reforms not been implemented. We used 3 different approaches to estimate this association using different counterfactuals.

Acute care hospitals, paid under the Inpatient Prospective Payment System, were subject to numerous payment reforms under the ACA. Within the population of acute care hospitals, we tested whether changes in spending were greater for commonly targeted diagnoses compared with untargeted diagnoses. This analysis excluded discharges for chronic obstructive pulmonary disease and lower extremity joint replacement. These diagnoses were included in the HRRP beginning in fiscal year 2015 and, therefore, did not fit clearly into the targeted and untargeted classification. By examining changes in spending among only acute care hospitals, this analysis addresses challenges associated with comparing spending across very different types of hospitals. At the same time, estimates from this specification could underestimate the changes associated with reforms following the ACA given that some provisions, such as Medicare fee cuts, apply to the broad spectrum of hospital care rather than the 3 common diagnoses that have been commonly been targeted in ACA programs. In addition, spillovers that reduced spending for untargeted diagnoses due to broad care improvements would also bias estimates from this approach. For this reason, estimates from this specification represent a lower bound of the association of the ACA with episode spending.

Second, we tested whether changes in spending were different between acute care and critical access hospitals. Critical access hospitals are small, rural hospitals that receive cost-based reimbursement (rather than prospective payment). These hospitals were exempt from nearly all ACA provisions targeting hospital spending. While critical access hospitals care for patients with many common medical conditions, including diagnoses targeted under the ACA, they differ from acute care hospitals across a range of characteristics, including size, rurality, and teaching status. The patients seen in critical access hospitals tend to have lower severity, and critical access hospitals tend to provide less intensive and technologically sophisticated care. However, recent work has shown that critical access hospitals perform similarly to acute care hospitals on important measures, including mortality rates for common surgical procedures.^26^ In addition, differences between acute care and critical access hospitals remained similar during the study period, limiting the bias associated with comparing outcomes across different populations.

For these 2 comparisons (targeted diagnoses vs untargeted diagnoses and acute care hospitals vs critical access hospitals), we performed difference-in-differences (DID) analyses, estimating linear regression models at the patient-episode level. We estimated separate models for total spending and each component of spending. Our models controlled for age, sex, race, season of admission, severity measured by the hierarchical condition category risk score, diagnosis (based on Agency for Healthcare Research and Quality clinical classification software),^27^ hospital characteristics (ie, size, teaching status, Medicaid share of patients, profit status), and hospital fixed effects (eAppendix in the Supplement). The DID models comparing acute care with critical access hospitals controlled for DRG weights instead of clinical classification. We considered the beginning of the reforms to be April 1, 2010, the month following the passage of the ACA. Budget sequestration began on March 1, 2013. We did not independently assess the association of budget sequestration with episode spending. A timeline of the relevant provisions occurring during the study period is included in eFigure 2 in the Supplement.

To evaluate the validity of our DID approach, we tested whether trends in the study outcomes were parallel for both DID analyses.^28^ When comparing spending for targeted and untargeted outcomes, trends were parallel with the exception of inpatient physician services (eTable 1 in the Supplement). When comparing spending between acute care and critical access hospitals, trends were also similar but statistically different for total episode spending and several components of episode spending (eTable 1 and eFigures 3-8 in the Supplement).

To address concerns that the challenges associated with the 2 DID approaches (including nonparallel trends) may lead to bias, we performed a third analysis using a generalized synthetic control approach.^29^ Originally developed for a single unit subject to treatment, the generalized synthetic control approach allows for multiple treated units (hospitals), as we have in our study.^29^ This approach was developed to identify a comparison group to minimize bias, particularly in cases when parallel trends were violated and the researcher had numerous untreated units that could potentially serve as controls.^24^ Under this approach, an interactive fixed-effect model is estimated by using control group data. Then, estimates of factor loadings for each treated unit are used to impute the counterfactual for the treatment group (ie, the value of the outcome for the treatment group in the postperiod if they did not receive treatment). Because panel data are required for synthetic control methods, we performed the generalized synthetic control analysis at the hospital-quarter level. We used mean age, sex, race, hierarchical condition category score, DRG weights, profit status of hospitals, and proportion of Medicare and Medicaid days as covariates in the model (eAppendix and eFigures 9-11 in the Supplement). We then specified the generalized synthetic control model to be created on the basis of episode spending in the 9 quarters prior to the start of the ACA.

We performed a supplemental analysis to approximate the contribution of Medicare’s fee reductions to hospital episode spending. Our analysis of changes in spending related to the index hospitalization is an approximation of Medicare fee reductions. However, this approximation may not be entirely accurate given that the index hospitalization includes outlier payments and could be affected by changes in the composition of DRGs over time. To address this, we created a hospital inpatient price index consisting of the 20 most common DRGs during the study period. We then performed a DID analysis, comparing changes in the price index between acute care and critical access hospitals (eAppendix and eTable 2 in the Supplement).

To test the robustness of our findings, we estimated models without accounting for coded severity of illness, which has been shown to inflate the association between other ACA reforms and outcomes.^12^ We also estimated models that did not standardize prices across facilities, and to reduce the influence of outliers, Winsorized spending at the 5th and 95th percentiles. Finally, because many of the reforms in the ACA focused on traditional Medicare (rather than Medicare Advantage), the association of the ACA with outcomes may have been stronger in areas with more traditional Medicare enrollment. To explore this, we estimated the association between reforms following the ACA and spending among hospitals in Hospital Referral Regions that had the highest and lowest Medicare Advantage penetration.

We used our estimates to approximate the annual savings to Medicare that resulted from reforms following the ACA. To do this, we multiplied our effect estimates by the number of annual episodes to which the estimate applied in the postreform period.

All reported *P* values were 2-sided, and *P* < .05 was used as threshold for significance. DID analyses were performed with Stata version 15 (StataCorp). The generalized synthetic control models were estimated using the gsynth package in R version 3.6.3 (R Project for Statistical Computing).^30^ Analysis of this data was initiated February 1, 2019 and completed on July 8, 2020.

## Results

A total of 7 634 242 episodes (4 525 630 [59.2%] female patients; mean [SD] age, 79.31 [8.02] years) from Medicare beneficiaries were included in this study. At acute care hospitals in the prereform period, 1 354 053 of 2 278 544 discharges (59.4%) were among female patients; the mean (SD) age of patients was 79.2 (7.8) years. At critical access hospitals in the prereform period, 63 983 of 102 564 discharges (62.4%) were among female patients; the mean (SD) age of patients was 80.9 (8.1) years (Table 1). The composition of patients at acute care and critical access hospitals changed little during the reform period.

**Table 1.** Patient and Hospital Characteristics Before and After Passage of the Patient Protection and Affordable Care Act^a^

Characteristic	Prereform, January 2008-March 2010		Postreform, April 2010-August 2015	
	Critical access hospital (n = 102 564)	Acute care hospital (n = 2 278 544)	Critical access hospital (n = 210 2010	Acute care hospital (n = 5 042 933)
Age, mean (SD), y	80.92 (8.07)	79.24 (7.84)	80.88 (8.26)	79.25(8.07)
Women	63 983 (62.4)	1 354 053 (59.4)	130 462 (62.1)	2 977 132 (59.0)
White race	96 612 (94.2)	1 991 226 (87.4)	198 260 (94.3)	4 382 927 (86.9)
Prevalence of targeted conditions				
AMI	863 (0.8)	60 183 (2.6)	1855 (0.9)	137 877 (2.7)
Heart failure	7306 (7.1)	127 343 (5.6)	14 325 (6.8)	273 040 (5.4)
Pneumonia	14 240 (13.9)	130 912 (5.7)	29 546 (14.1)	278 637 (5.5)
30-d readmission rate, mean (SD)				
All conditions	13.98 (0.35)	14.47 (0.35)	13.08 (0.34)	13.63 (0.34)
AMI	17.50 (0.38)	18.52 (0.39)	15.58 (0.36)	16.52 (0.37)
Heart failure	19.92 (0.40)	21.23 (0.41)	17.28 (0.38)	19.93 (0.40)
Pneumonia	13.01 (0.34)	15.35 (0.36)	12.71 (0.33)	14.45 (0.35)
HCC score, mean (SD)	1.55 (1.05)	1.57 (1.20)	1.68 (1.17)	1.76 (1.35)
DRG weight	0.96 (0.45)	1.49 (1.27)	1.01 (0.50)	1.55 (1.29)
Bed size				
<200	102 564 (100.0)	687 139 (30.2)	210 201 (100.0)	1 499 995 (29.7)
200-349	0	673 805 (29.6)	0	1 453 347 (28.8)
350-499	0	412 378 (18.1)	0	902 060 (17.9)
≥500	0	505 222 (22.2)	0	1 187 531 (23.5)
Profit status				
For-profit	5134 (5.0)	329 469 (14.5)	10 885 (5.2)	797 392 (15.8)
Nonprofit	59 416 (57.9)	1 693 771 (74.3)	124 808 (59.4)	3 716 955 (73.7)
Other	38 014 (37.1)	255 304 (11.2)	74 508 (35.4)	528 586 (10.5)
Geographic region				
Midwest	51 108 (49.8)	571 034 (25.1)	99 706 (47.4)	1 227 230 (24.3)
Northeast	8125 (7.9)	468 614 (20.6)	17 686 (8.4)	1 019 524 (20.2)
South	26 939 (26.3)	905 995 (39.8)	55 208 (26.3)	2 021 241 (40.1)
West	16 392 (16.0)	332 901 (14.6)	37 601 (17.9)	774 938 (15.4)
Teaching hospitals	0	391 315 (17.2)	0	859 800 (17.0)
Urban location	39 550 (38.6)	2 213 243 (97.1)	83 289 (39.6)	4 922 828 (97.6)
Medicaid days, mean (SD), %	18.27 (0.21)	17.17 (0.10)	17.55 (0.18)	18.54 (0.10)
Medicare days, mean (SD), %	54.10 (0.23)	50.84 (0.12)	55.59 (0.26)	51.27 (0.12)

Abbreviations: AMI, acute myocardial infarction; DRG, diagnosis-related group; HCC, hierarchical condition category.

^a^ Cohort size is based on episodes included in difference-in-differences analysis comparing acute care and critical access hospitals for all diagnoses.

For DID comparisons between targeted and untargeted diagnoses among acute care hospitals, our sample included 1 007 992 episodes from patients discharged with the targeted diagnoses and 5 543 555 episodes from patients with the untargeted diagnoses (3502 unique hospitals). For DID comparisons between acute care and critical access hospitals, our sample included 7 321 477 episodes (1 007 992 with targeted diagnoses, 5 543 555 with untargeted diagnoses, and 769 930 with eventually targeted diagnoses) from patients discharged from 3505 acute care hospitals and 312 765 episodes (68 135 with targeted diagnoses, 210 288 with untargeted diagnoses, and 34 342 with eventually targeted diagnoses) from patients discharged from 1354 critical access hospitals. The sample for the generalized synthetic control comparisons between acute care and critical access hospitals was somewhat smaller because the analysis required at least 6 quarters of data during the preintervention period.

Table 1 shows the characteristics of patients admitted to acute care and critical access hospitals in the prereform and postreform periods. Compared with critical access hospitals in the prereform period, acute care hospitals had a lower share of admissions for White patients (96 612 [94.2%] vs 1 991 226 [87.4%]), a higher share of admissions for acute myocardial infarction (863 [0.8%] vs 60 183 [2.6%]), and a lower share of admissions for pneumonia (14 240 [13.9%] vs 130 912 [5.7%]). Additionally, 30-day readmission rates and the number of hierarchical condition categories (capturing patient risk) were somewhat higher for acute care hospitals than for critical access hospitals (mean [SD] 30-d readmission rates for all conditions: 14.47 [0.35] vs 13.98 [0.35]; mean [SD] hierarchical condition categories score: 1.57 [1.20] vs 1.55 [1.05]). Finally, acute care hospitals were much larger and more likely to be teaching hospitals. However, the differences between acute care and critical access hospitals remained similar in the postreform period. This supports the validity of the DID approach, which holds constant all factors that do not change across the prereform and postreform periods.

In the prereform period, total episode spending was greater for acute care hospitals and among targeted diagnoses. For instance, in the DID comparisons between acute care and critical access hospitals, mean (SD) total episode spending for all diagnoses was $15 034 ($14 710) for acute care hospitals and $12 194 ($10 832) for critical access hospitals; mean (SD) total episode spending for targeted diagnoses was $15 007 ($14 591) for acute care hospitals and $12 754 ($10 488) for critical access hospitals; and mean (SD) total episode spending for untargeted diagnoses was $15 013 ($15 179) for acute care hospitals and $12 073 ($11 121) for critical access hospitals (eTable 3 in the Supplement).

The Figure shows time series data for the different comparisons. Figure, A shows that episode spending decreased consistently more for targeted diagnoses compared with untargeted diagnoses after the reforms were initiated. Figure, B shows that spending decreased more for acute care hospitals relative to the full sample of critical access hospitals. Figure, C shows spending decreased for acute care hospitals relative to the synthetic control hospitals. Figure, B and Figure, C indicate that differences in spending between acute care and critical access hospitals tended to grow during the period after the reforms were implemented.

**Figure.**
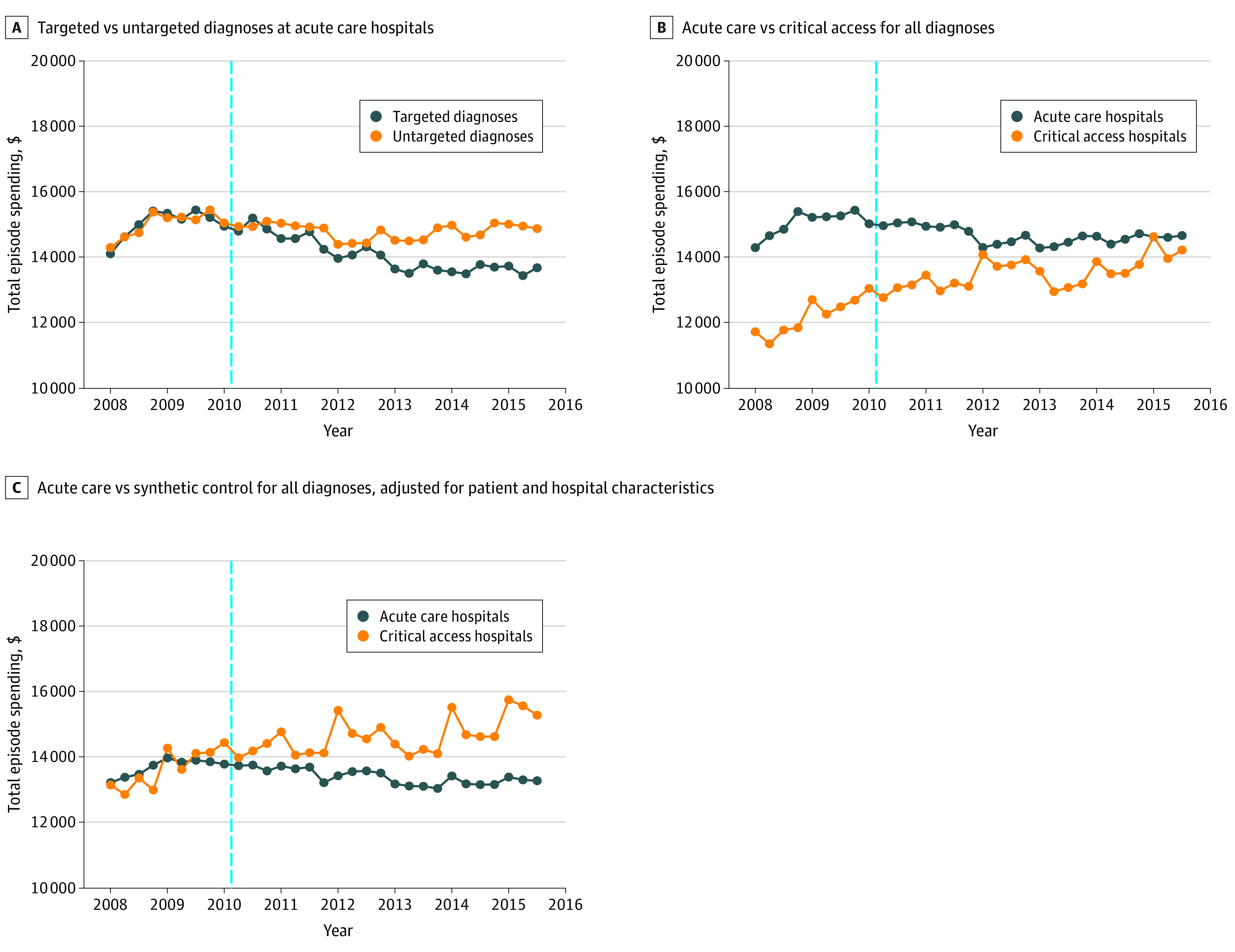
Total Episode Spending Before and After Reforms Beginning With the Passage of the Patient Protection and Affordable Care Act

Table 2 shows the alternative estimates of the association between the reforms and episode spending across the hospital populations, statistical comparisons, and the patient populations. The DID estimate comparing targeted and untargeted diagnoses among acute care hospitals was −$431 (95% CI, −$492 to −$369). Dividing this estimate by the preintervention mean for the comparison group ($15 013) suggests a 2.87% reduction in episode spending. The DID estimate comparing acute care and critical access hospitals for all diagnoses was −$1820 (95% CI, −$1897 to −$1743). This estimate was similar for targeted diagnoses (−$1957; 95% CI, −$2199 to −$1743) and untargeted diagnoses (−$1949; 95% CI, −$2045 to −$1853). Estimates from the generalized synthetic control models were smaller than the DID estimates for all diagnoses (−$1232; 95% CI, −$1488 to −$956), targeted diagnoses (−$995; 95% CI, −$1197 to −$781), and untargeted diagnoses (−$1147; 95% CI, −$1400 to −$734). The generalized synthetic control model estimate amounts to a 10.12% reduction in episode spending.

**Table 2.** Estimates of Association Between Reforms Following the Patient Protection and Affordable Care Act and Hospital Episode Spending^a^

Hospital population and comparison	Patient population	No.	Effect estimate (95% CI), $
Acute care only			
DID, targeted vs untargeted diagnoses	All diagnoses	6 551 547	−431 (−492 to −369)
**Acute care and critical access**			
DID, acute care vs critical access	All diagnoses	7 634 242	−1820 (−1897 to −1743)
	Targeted diagnoses	1 076 127	−1957 (−2199 to −1743)
	Untargeted diagnoses	5 753 843	−1949 (−2045 to −1853)
**Acute care and critical access**			
Generalized synthetic	All diagnoses	7 566 671	−1232 (−1488 to −965)
Control, acute care vs critical access	Targeted diagnoses	1 058 831	−995 (−1197 to −781)
	Untargeted diagnoses	5 702 998	−1147 (−1400 to −734)

Abbreviations: DID, difference-in-differences.

^a^ 95% CIs for DID models are based on standard errors that are robust to clustering at hospital level. Standard errors for generalized synthetic control models were estimated using bootstrapping with 1000 iterations. Targeted diagnoses include acute myocardial infarction, heart failure, and pneumonia. Discharges for chronic obstructive pulmonary disease and lower extremity joint replacement were excluded from acute care only DID models. Models estimated among targeted diagnoses controlled for whether targeted condition was acute myocardial infarction, heart failure, or pneumonia.

Results from models examining the association between reforms following the ACA and components of episode spending are shown in Table 3. Estimates show that the largest reductions were related to the index admission, followed by spending related to readmissions and postacute care. Supplemental analysis found that spending reductions related to the index admission were largely associated with cuts to Medicare fees (eTable 4 in the Supplement).

**Table 3.** Estimates of Association Between Reforms Following the Patient Protection and Affordable Care Act and Components of Episode Spending for All Diagnoses^a^

Spending component	Effect estimate (95% CI), $		
	DID		Generalized synthetic control, acute care vs critical access hospitals (n = 7 566 671)
	Targeted vs untargeted diagnoses (n = 6 551 547)	Acute care vs critical access hospitals (n = 7 634 242)	
Index hospitalization, facility services	−331 (−362 to −300)	−1162 (−1182 to −1142)	NA
Physician services			
Inpatient	−26 (−32 to −20)	−30 (−37 to −22)	−20 (−40 to −7)
Outpatient	7 (0 to 13)	14 (4 to 25)	193 (180 to 206)
Facility services			
Readmissions	−49 (−74 to −24)	−154 (−195 to −113)	−243 (−323 to −176)
Hospital outpatient	−16 (−21 to −11)	−120 (−128 to −111)	155 (119 to 226)
Post-acute care	−15 (−44 to 13)	−370 (−418 to −321)	−173 (−370 to −43)

Abbreviations: DID, difference-in-differences; NA, not available.

^a^ 95% CIs for DID models are based on standard errors that are robust to clustering at hospital level. Standard errors for generalized synthetic control models were estimated using bootstrapping with 1000 iterations. NA indicates that the estimate was not available due to failure of model to converge (eAppendix in the Supplement). Targeted diagnoses include acute myocardial infarction, heart failure, and pneumonia. Discharges for chronic obstructive pulmonary disease and lower extremity joint replacement were excluded from acute care only DID models. Models estimated among targeted diagnoses controlled for whether targeted condition was acute myocardial infarction, heart failure, or pneumonia.

Table 4 shows that the estimates were similar for models that did not use standardized prices and did not adjust for coded patient severity (ie, hierarchical condition categories). However, estimates were larger in regions with lower Medicare Advantage penetration: for instance, the estimate from generalized synthetic control models in regions in the highest tercile of Medicare Advantage penetration was −$929 (95% CI, −$1272 to −$591) compared with −$1433 (95% CI, −$1898 to −$1064) in regions in the lowest tercile of Medicare Advantage penetration.

**Table 4.** Estimates of Association Between Reforms Following the Patient Protection and Affordable Care Act and Total Hospital Spending for All Diagnoses From Alternative Specifications

Specification	DID		Generalized synthetic control, acute care vs critical access hospitals
	Targeted vs untargeted diagnoses^**a**^	Acute care vs critical access hospitals^**b**^	
**No price standardization**			
No.	6 551 547	7 634 242	7 566 671
Treatment effect estimate (95% CI), $	−418 (−494 to −343)	−1947 (−2042 to −1852)	−1978 (−2279 to −1683)
**No adjustment for coded risk**			
No.	6 551 547	7 634 242	7 566 671
Treatment effect estimate (95% CI), $	−398 (−459 to −337)	−1781 (−1859 to −1704)	−1214 (−1458 to −946)
**Hospitals in regions with highest tercile of Medicare Advantage penetration**			
No.	2 190 772	2 562 193	2 476 099
Treatment effect estimate (95% CI), $	−382 (−524 to −240)	−1562 (−1774 to −1350)	−929 (−1272 to −591)
**Hospitals in regions with lowest tercile of Medicare Advantage penetration**			
No.	1 890 385	2 268 390	2 254 501
Treatment effect estimate (95% CI), $	−485 (−616 to −354)	−2164 (−2298 to −2030)	−1433 (−1898 to −1064)
**Winsorized payment**			
No.	6 551 547	7 634 242	7 566 671
Treatment effect estimate (95% CI), $	−470 (−511 to −430)	−1413 (−1473 to −1353)	−995 (−1189 to −793)

Abbreviation: DID, difference-in-differences.

^a^ All targeted vs untargeted diagnoses models were adjusted for age, race, sex, hierarchical condition category score, clinical classifications software, hospital profit status, proportion of Medicare days, and proportion of Medicaid days.

^b^ All acute care vs critical access hospitals DID models were adjusted for age, race, sex, hierarchical condition category score, diagnosis-related group weight, hospital profit status, proportion of Medicare days, and proportion of Medicaid days.

Estimates from the generalized synthetic control model (−$1232), applied to 4.99 million annual episodes occurring in acute care hospitals during the study period, imply that reforms following the ACA reduced Medicare hospital episode spending by $5.68 billion annually (eTable 5 in the Supplement). Estimates of total savings to Medicare range from $274 million annually (DID estimates comparing targeted and untargeted diagnoses among acute care hospitals) to $8.47 billion annually (DID estimates comparing acute care and critical access hospitals for all diagnoses).

## Discussion

Our study has 3 principal findings that improve our understanding of reforms following the ACA. First, across several alternative estimation strategies, reforms following the ACA were associated with substantial reductions in hospital episode spending. Second, reductions in episode spending were the greatest for the index admission and readmission hospitalization spending. The substantial reductions in spending for the index admission coupled with other evidence suggests that Medicare fee reductions were associated with most of the reductions in episode spending. Third, spending reductions were substantially greater in areas with lower Medicare Advantage penetration.

To our knowledge, ours is the first study to evaluate whether reforms following the ACA were associated with reductions in hospital episode spending. Our findings are consistent with national data showing virtually no growth in hospital inpatient spending following the ACA^31^ and greater spending growth for critical access hospitals compared with acute care hospitals.^32^ A number of studies have assessed whether specific policies implemented or authorized under the ACA, including the HRRP,^2,11^ Hospital Value-Based Purchasing,^1^ and Bundled Payment for Care Improvement,^8,13^ achieved reduced spending or improved quality. Estimates from the Congressional Budget Office (CBO) indicate that the ACA and budget sequestration led to reductions of Medicare spending between $6.7 and $12.1 billion annually between 2010 and 2015 (eTable 6 in the Supplement). This is consistent with our estimate of $5.68 billion in annual savings, which included only savings related to hospital episode spending.

Also consistent with CBO estimates, the balance of evidence suggests that Medicare reimbursement cuts were the largest factor associated with Medicare spending reductions observed in our study. The substantial role of fee reductions in changes in episode spending can be seen in our study by: (1) large association of reforms following the ACA with spending related to the index admission (which is not affected by changes in postdischarge utilization); (2) large association of reforms following the ACA with a hospital price index; (3) smaller changes associated with reforms based on comparisons between targeted and untargeted diagnoses (which are both subject to Medicare fee reductions); (4) the fact that critical access hospitals were not subject to Medicare fee reductions under the ACA^33^ (critical access hospitals were subject to fee cuts under sequestration; because critical access hospitals are paid under a cost-based reimbursement system, these hospitals could counteract Medicare fee cuts by increasing reported costs); and (5) greater differences emerging between treatment and comparison groups beginning in 2012 (Figure) when additional provisions cutting Medicare fees began to take effect (eFigure 8 in the Supplement). Our conclusion about the relative importance of fee cuts is supported by CBO estimates of sequestration cuts of Medicare payments and ACA reductions in the annual updates to Medicare’s payment rates, explaining most of the estimated Medicare savings during this time period, with changes such as the Medicare Shared Savings Program explaining a much smaller portion (eTable 6 in the Supplement).

Reductions in readmission rates and in the use of postacute care after discharge likely also contributed to savings. However, although we did not estimate this directly, Medicare fee reductions would also contribute to the associations of reforms with spending related to readmissions. As a result, we conclude that reforms following the ACA were associated with reductions in hospital episode spending, primarily through Medicare fee reductions for hospitals and through reductions in spending for postacute care.

### Limitations

Our study should be interpreted in the context of several important limitations. First, we were not able to evaluate the association of reforms following the ACA with spending using an ideal control group, ie, hospitals that were similar to acute care hospitals but not exposed to reforms. Instead, our estimates used a combination of untargeted diagnoses and critical access hospitals (not exposed to ACA reforms) as the counterfactual. Nonetheless, across a wide range of estimation strategies, we found consistent evidence that reforms following the ACA were associated with substantial reductions in spending.

Second, by using administrative data, we cannot fully account for patient risk, which can drive spending patterns. However, using administrative data sources allows us to closely adhere to the same data used to administer and set penalties under Medicare reforms.^34^ A sensitivity analysis found that our results were not sensitive to comorbidity coding. Third, because we did not have access to the data, our study did not evaluate Medicare spending associated with home health care and durable medical equipment.^35^ Because the CBO estimated that the ACA led to $0.75 billion in annual reductions related to home health spending during our study period, our overall estimates may be understated.^17^ In addition, our analysis of total spending reductions assumed that the reforms we considered had no effect on the volume of admissions in the postreform period. Quarterly volume declined for acute care hospitals from approximately 253 000 in the prereform period to 235 000 in the postreform period. ACA reforms focusing on efficiency could have contributed to these reductions. At the same time, hospitals may have tried to increase volume to counteract fee cuts. Because the magnitude of these countervailing effects are ambiguous, we did not consider them in our estimates of total spending reductions.

Fourth, we examined hospital spending within 30 days of discharge, rather than the 90-day period now commonly used in bundled payment programs. However, 30-day episode spending is commonly used in national programs, such as Hospital Compare^36^ and Hospital Value-Based Purchasing. In addition, between 75% and 84% of 90-day episode spending occurs within a 30-day episode.^37^ For these reasons, 30-day episodes are appropriate to assess hospital spending, and the use of longer episode durations are unlikely to affect our conclusions.

Fifth, while a longer preintervention period would have enhanced the validity of our study design, Medicare’s transition to Medicare Severity–DRG (MS-DRG) on October 1, 2007, precluded our ability to accurately compare spending across similar admissions before and after the initiative of MS-DRGs. Sixth, our evaluation did not decompose pure price and quantity effects of reforms following the ACA. Our assessment of changes in prices only considered hospital inpatient prices. Medicare fee reductions for physician, hospital outpatient, and postacute care services also contributed to reductions in episode spending, although we did not estimate the precise magnitude of these fee reductions. Seventh, given the many myriad changes that occurred in US health care around the time of the ACA, we are limited in our ability to determine the precise mechanism that led to reductions in Medicare spending.

## Conclusions

This policy evaluation has important implications for stakeholders seeking to understand the impact of policies to reduce spending in Medicare. This study found that a combination of provisions in the ACA and budget sequestration, likely associated with overall payment reductions and penalties as well as reforms focused on quality improvement, were associated with reductions in Medicare spending. This suggests that multipronged policy changes focused on conditional or unconditional payment reductions can generate substantial savings.

## Notes

Supplement. **eAppendix. **Supplementary Methods **eFigure 1. **Sample Flow Diagram **eFigure 2.** Timeline of Key Budget Control Act and Affordable Care Act Programs and Policies **eFigure 3. **Time Series: Spending for Index Admission, 2008-2015 **eFigure 4. **Time Series: Spending for Inpatient Physician Services, 2008-2015 **eFigure 5. **Time Series: Spending for Outpatient Physician Services, 2008-2015 **eFigure 6. **Time Series: Spending for Readmissions (Hospital Component Only), 2008-2015 **eFigure 7. **Time Series: Spending for Postacute Care, 2008-2015 **eFigure 8. **Time Series: Spending for Hospital Outpatient Care, 2008-2015 **eFigure 9. **Latent Factor Values From Generalized Synthetic Model **eFigure 10. **Factor Loadings From Generalized Synthetic Control Model **eFigure 11. **Implausible Counterfactual Estimates From Generalized Synthetic Control Model Estimated for Index Admission Spending **eTable 1. **Parallel Trends Tests Between Targeted and Untargeted Outcomes and Between Acute Care and Critical Access Hospitals **eTable 2. **DRGs Included in Price Index **eTable 3. **Levels of Preintervention Spending for Statistical Comparisons of Association Between ACA and Hospital Episode Spending **eTable 4. **Difference-in-Differences Analysis of Changes in Hospital Prices Following Reforms **eTable 5. **Estimated Annual Changes in Spending by Medicare After the ACA by Payment Component **eTable 6. **Analysis of CBO Estimates in Billions of Dollars
