# Supplementary material for: Evaluation of US Hospital Episode Spending for Acute Inpatient Conditions After the Patient Protection and Affordable Care Act
Source: JAMA Netw Open. 2020 Nov 23;3(11):e2023926. doi: 10.1001/jamanetworkopen.2020.23926 (PMC7684450; doi:10.1001/jamanetworkopen.2020.23926)
Supplement: Supplement. — eAppendix. Supplementary Methods eFigure 1. Sample Flow Diagram eFigure 2. Timeline of Key Budget Control Act and Affordable Care Act Programs and Policies eFigure 3. Time Series: Spending for Index Admission, 2008-2015 eFigure 4. Time Series: Spending for Inpatient Physician Services, 2008-2015 eFigure 5. Time Series: Spending for Outpatient Physician Services, 2008-2015 eFigure 6. Time Series: Spending for Readmissions (Hospital Component Only), 2008-2015 eFigure 7. Time Series: Spending for Postacute Care, 2008-2015 eFigure 8. Time Series: Spending for Hospital Outpatient Care, 2008-2015 eFigure 9. Latent Factor Values From Generalized Synthetic Model eFigure 10. Factor Loadings From Generalized Synthetic Control Model eFigure 11. Implausible Counterfactual Estimates From Generalized Synthetic Control Model Estimated for Index Admission Spending eTable 1. Parallel Trends Tests Between Targeted and Untargeted Outcomes and Between Acute Care and Critical Access Hospitals eTable 2. DRGs Included in Price Index eTable 3. Levels of Preintervention Spending for Statistical Comparisons of Association Between ACA and Hospital Episode Spending eTable 4. Difference-in-Differences Analysis of Changes in Hospital Prices Following Reforms eTable 5. Estimated Annual Changes in Spending by Medicare After the ACA by Payment Component eTable 6. Analysis of CBO Estimates in Billions of Dollars [file jamanetwopen-e2023926-s001.pdf]

Supplemental Online Content

Ibrahim AM, Nuliyalu U, Lawton EJ, et al. Evaluation of US hospital episode spending for acute inpatient conditions after the Patient Protection and Affordable Care Act. *JAMA Netw Open*. 2020;3(11):e2023926. doi:10.1001/jamanetworkopen.2020.23926

eAppendix. Supplementary Methods
eFigure 1. Sample Flow Diagram
eFigure 2. Timeline of Key Budget Control Act and Affordable Care Act Programs and Policies
eFigure 3. Time Series: Spending for Index Admission, 2008-2015
eFigure 4. Time Series: Spending for Inpatient Physician Services, 2008-2015
eFigure 5. Time Series: Spending for Outpatient Physician Services, 2008-2015
eFigure 6. Time Series: Spending for Readmissions (Hospital Component Only), 2008-2015
eFigure 7. Time Series: Spending for Postacute Care, 2008-2015
eFigure 8. Time Series: Spending for Hospital Outpatient Care, 2008-2015
eFigure 9. Latent Factor Values From Generalized Synthetic Model
eFigure 10. Factor Loadings From Generalized Synthetic Control Model
eFigure 11. Implausible Counterfactual Estimates From Generalized Synthetic Control Model Estimated for Index Admission Spending
eTable 1. Parallel Trends Tests Between Targeted and Untargeted Outcomes and Between Acute Care and Critical Access Hospitals
eTable 2. DRGs Included in Price Index
eTable 3. Levels of Preintervention Spending for Statistical Comparisons of Association Between ACA and Hospital Episode Spending
eTable 4. Difference-in-Differences Analysis of Changes in Hospital Prices Following Reforms
eTable 5. Estimated Annual Changes in Spending by Medicare After the ACA by Payment Component
eTable 6. Analysis of CBO Estimates in Billions of Dollars

This supplemental material has been provided by the authors to give readers additional information about their work.

© 2020 Ibrahim AM et al. *JAMA Network Open.*

eAppendix. Supplementary Methods

Estimating Equations for Difference-in-differences

Our difference-in-difference estimator comparing targeted and untargeted diagnoses included patient covariates, hospital covariates, in indicator for the season of admission, an indicator for exposed hospitals, an indicator for the post-intervention period (beginning April 1, 2010), and an interaction between the treatment and post-intervention indicators. For episode *i* for diagnoses *j* in quarter *t*, we estimated the following equation:

$$(1)\quad Spending_{ijt} = b_0 + b_1 X_{ijt} + b_2 Z_{jt} + b_3\, season_t + b_4\, targeted_j + b_5\, post_t + b_6\, (targeted_j * post_t) + u_i + e_{ijt}$$

Where *X* is a vector of patient severity adjusters (age, gender, race, HCC score), *Z* is a vector of hospital characteristics (size, teaching status, share of Medicaid patients, profit status), *season* is a vector of season indicators, *targeted* indicates whether patients' index admission was a commonly targeted medical condition (AMI, heart failure, pneumonia), and *post* indicates whether the discharge occurred in the pre or post ACA period. b_6 is the difference-in-differences estimator. The equation was estimated separately for each study outcome.

Our difference-in-difference estimator comparing acute care and critical access hospitals took a similar form:

$$(2)\quad Spending_{ijt} = b_0 + b_1 X_{ijt} + b_2 Z_{jt} + b_3\, season_t + b_5\, post_t + b_6\, (acute\ care_j * post_t) + u_i + e_{ijt}$$

In this specification, the vector X also included admitting diagnoses (as captured by AHRQ clinical classification software).

Specifying the generalized synthetic control model

To address concerns that the challenges associated with the two DID approaches (including non-parallel trends) may lead to bias, we performed a third analysis using a generalized synthetic control approach.29 Originally developed for a single unit subject to treatment, the generalized synthetic control approach allows for multiple treated units (hospitals), as we have in our study. This approach was developed to identify a comparison group to minimize bias, particularly in cases when parallel trends were violated and the researcher had numerous untreated units that could potentially serve as controls. Under this approach, an interactive fixed effect model is estimated by using control group data. Then, estimates of factor loadings for each treated unit are used to impute the counterfactual for the treatment group (i.e., the value of the outcome for the treatment group in the post period if they did not receive treatment). Because panel data is required for synthetic control methods, we performed the generalized synthetic control analysis at the hospital-quarter level. We used average age, gender, race, HCC score, DRG weights, profit status of hospitals, and proportion of Medicare and Medicaid days as covariates in the model. We then specified the generalized synthetic control model to be created on the basis of episode spending in the 9 quarters prior to the start of the ACA.

We specified the generalized synthetic control model to include quarter effects but no hospital fixed effects. The generalized synthetic control model creates time-varying coefficients to derive counterfactual estimates for hospitals. These are known as latent factors, and the value of the hospital intercepts are factor loadings.

© 2020 Ibrahim AM et al. *JAMA Network Open.*

The model for overall spending, when optimized using cross-validation, returned a single factor. The value of the latent factor over the study period is shown in Appendix Figure 9. The y-axis represents the magnitude of the factor, although not directly interpetable in the units of the outcome (30-day spending). This figure shows the value of the factor declining over time. The values of the factor loadings are shown in Appendix Figure 10. Factor loadings represent the hospital-specific intercept values. While the values for acute care hospitals are higher than those for critical access hospitals, there is substantial overlap in the distribution.

We estimated separate generalized synthetic control models for each component of spending (shown in Table 3). For the index admission spending, the generalized synthetic model experienced problems with convergence and was not able to yield plausible counterfactual estimates, denoted y(0) in Appendix Figure 11. As a result, this model was not able to yield plausible estimates of the treatment effect. We did not report estimates from this model in Table 3.

Description of creating price index and evaluating changes in prices

We identified the 20 most common DRGs for acute care and critical access hospitals (combined) over the study period. We then performed a difference-in-difference analysis, comparing changes in the price index between acute Care and critical access hospitals for each of the DRG. Among these DRGs, we then calculated the share of admissions for each DRG over the study period. We then estimated the effect of reforms following the ACA on an index of hospital prices by multiplying the effects for a given DRG by that DRG's share of admissions over the study period. This analysis was performed using standardized and unstandardized Medicare payments.

Analysis of CBO Estimates for Comparison with Study Estimates

To compare our estimates of spending reductions with those from CBO, we reviewed CBO analyses of the Budget Control Act of 2011 and the Affordable Care Act.[1–3] Because estimates of budget sequestration cuts and ACA reductions in the annual updates to Medicare's payment rates apply not only to hospitals but also other providers, we separately estimated the share of each that is attributed to hospitals.

For the estimate of the hospital portion of sequestration cuts, we drew upon a corresponding aha.org report estimate.[4] This estimate was determined by calculating two percent of baseline Medicare fee-for-service spending as projected by CBO for hospital inpatient and outpatient services, in addition to hospital-based skilled nursing and home health. Because this estimate was for the 2013-2021 time period, we then recalculated this savings estimate for our study period.

For the estimate of the hospital portion of ACA reductions in annual updates to Medicare's payment rates, we drew upon a CBO analysis of the ACA over our study period as well as CBO estimates from a repeal of the ACA cost analysis in a later time period. In contrast to the CBO analysis of the ACA over our study period, this subsequent CBO analysis separately estimated the hospital services portion and the total portion (including home health) of ACA reductions in the annual updates to Medicare's payment rates. Based on this, we then calculated this hospital services percentage and multiplied this by the total CBO estimate (including home health) of ACA reductions in the annual updates to Medicare's payment rates reported for our study period, to obtain an estimate of the hospital portion for our study period.

Appendix Table 6 shows estimates of Medicare costs and savings (in billions of dollars), based on CBO estimates. We combined estimates related to costs and savings under the Budget Control Act of 2011 and the 2010 Affordable Care Act, for all applicable providers and for hospital providers separately. We conclude that CBO estimates indicate average annual savings between $7 billion and $12 billion during our study period.

© 2020 Ibrahim AM et al. *JAMA Network Open.*

References

1. Congressional Budget Office. *Estimated Impact of Automatic Budget Enforcement Procedures Specified in the Budget Control Act*. https://www.cbo.gov/sites/default/files/112th-congress-2011-2012/reports/09-12-BudgetControlAct_0.pdf .
2. Congressional Budget Office. *H.R. 4872, Reconciliation Act of 2010 (Final Health Care Legislation)*. https://www.cbo.gov/sites/default/files/111th-congress-2009-2010/costestimate/amendreconprop.pdf.
3. Congressional Budget Office. *Letter to the Honorable John Boehner Providing an Estimate for H.R. 6079, the Repeal of Obamacare Act*. https://www.cbo.gov/sites/default/files/112th-congress-2011-2012/costestimate/43471-hr6079_0.pdf .
4. Dobson A, DaVanzo J, Haught R, Luu P. *Estimate of Federal Payment Reductions to Hospitals Following the ACA 2010-2028*. https://www.aha.org/system/files/2018-06/estimate-of-fed-payment-reductions-to-hospitals-following-aca-2010-2018-report.pdf .

© 2020 Ibrahim AM et al. *JAMA Network Open.*

eFigure 1. Sample Flow Diagram

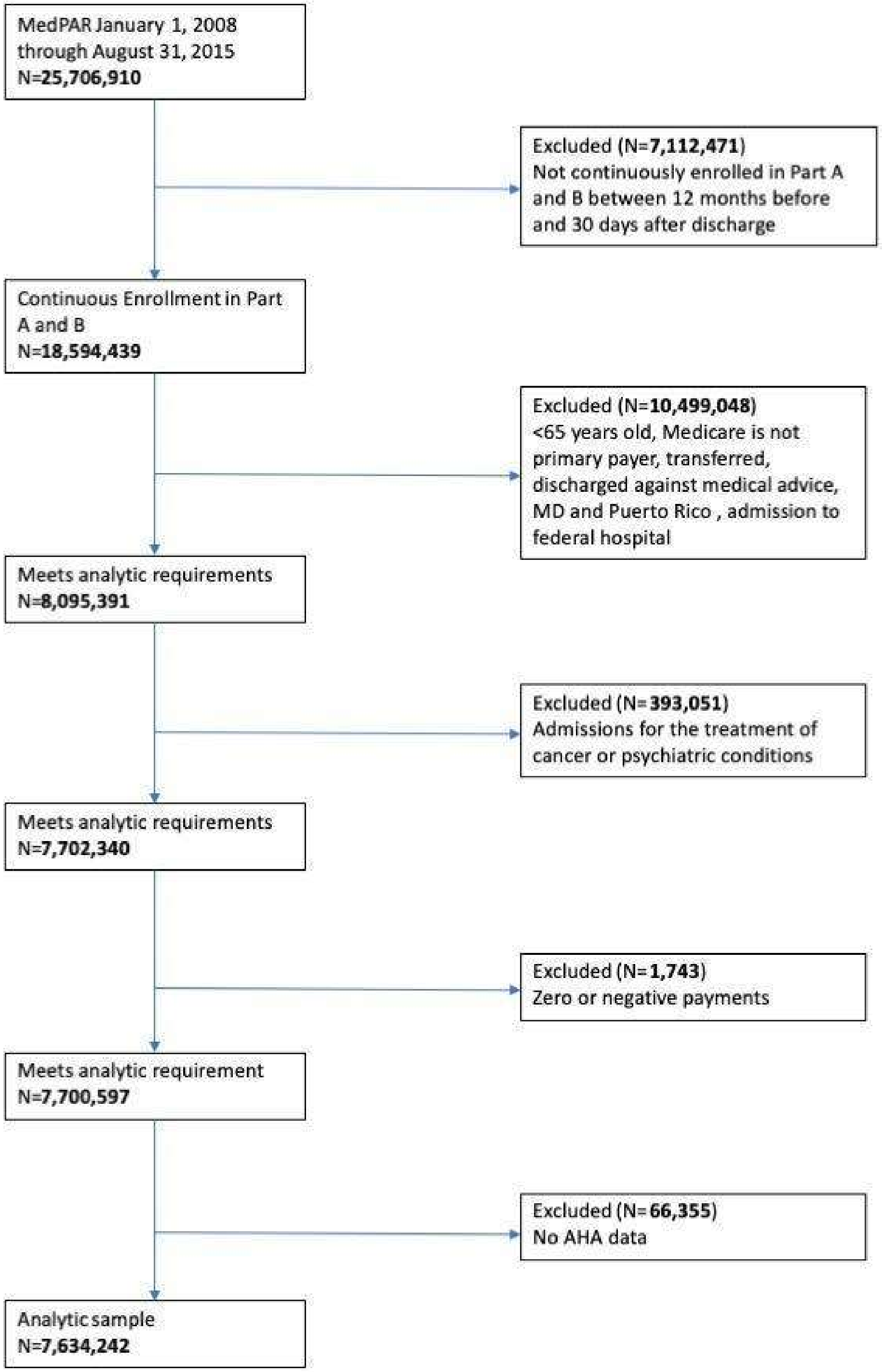

© 2020 Ibrahim AM et al. *JAMA Network Open.*

eFigure 2. Timeline of Key Budget Control Act and Affordable Care Act Programs and Policies

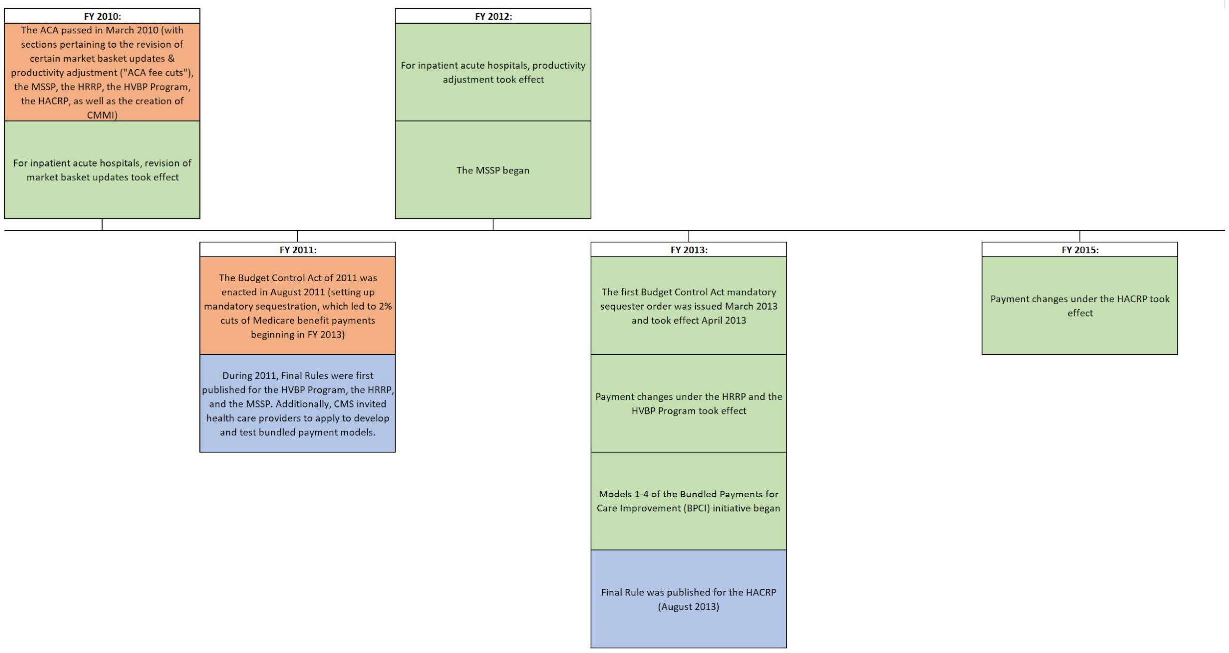

© 2020 Ibrahim AM et al. *JAMA Network Open.*

eFigure 3. Time Series: Spending for Index Admission, 2008-2015

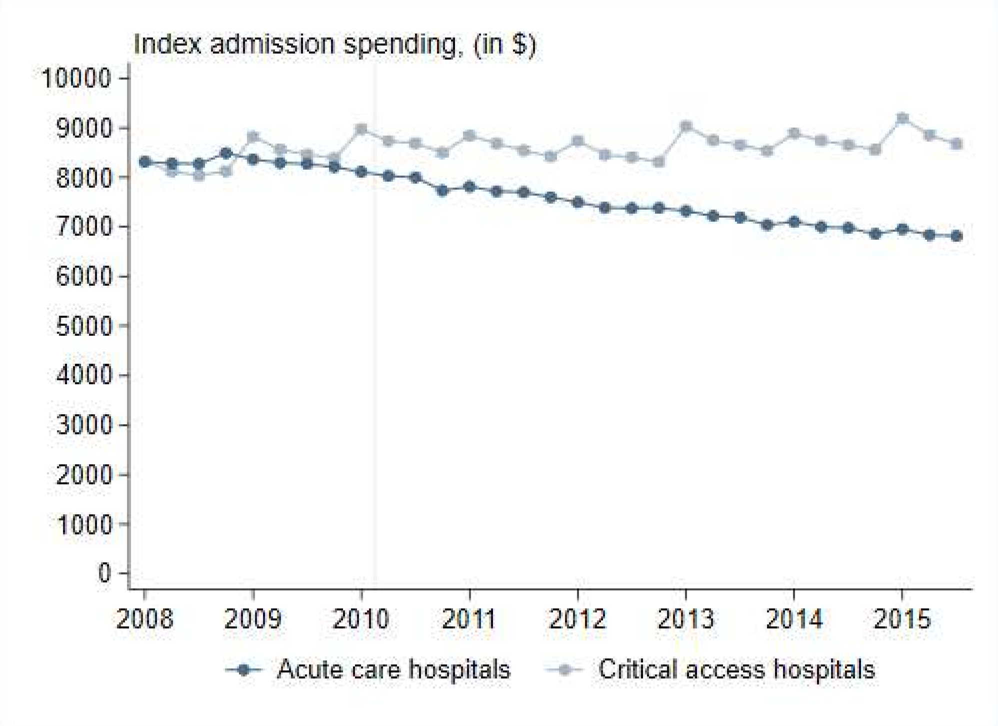

© 2020 Ibrahim AM et al. *JAMA Network Open.*

eFigure 4. Time Series: Spending for Inpatient Physician Services, 2008-2015

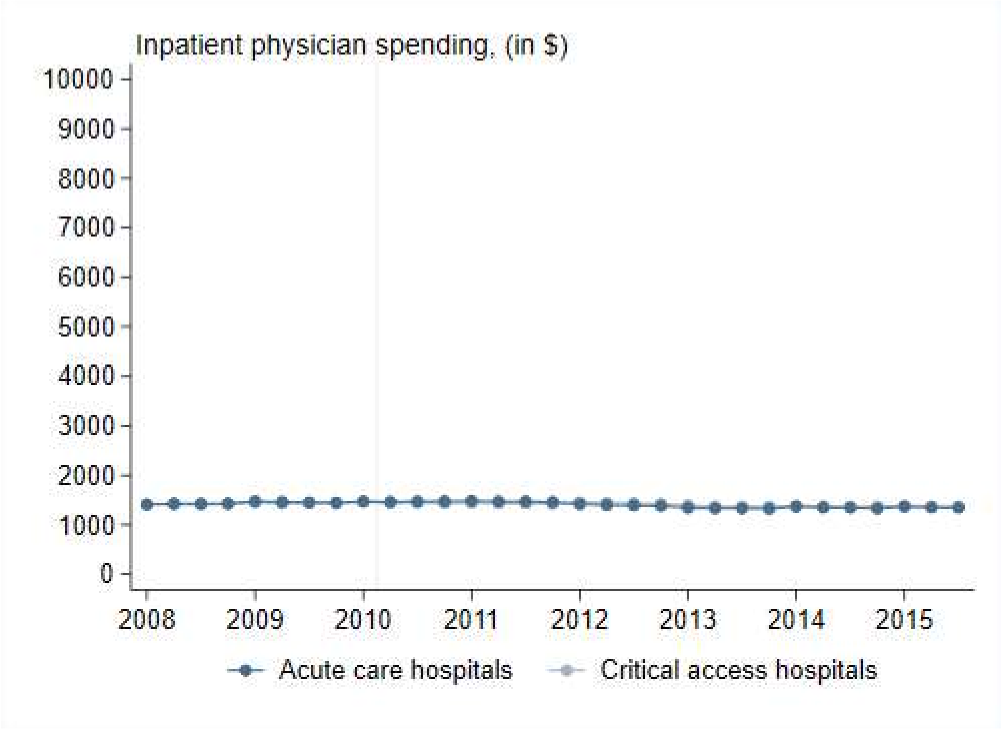

© 2020 Ibrahim AM et al. *JAMA Network Open.*

eFigure 5. Time Series: Spending for Outpatient Physician Services, 2008-2015

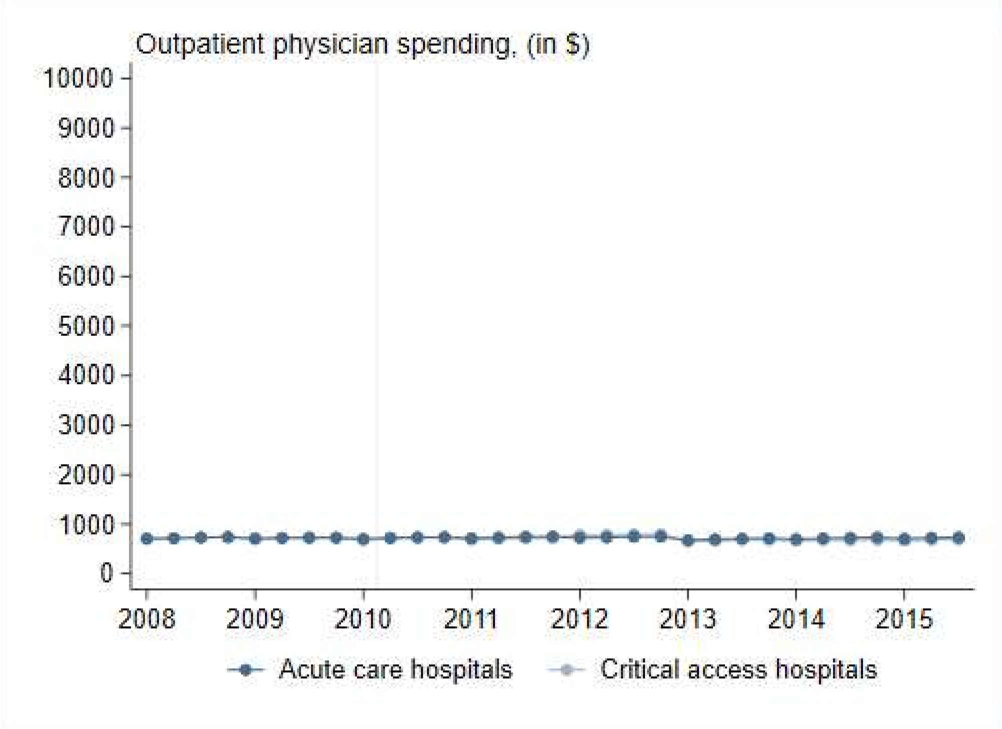

© 2020 Ibrahim AM et al. *JAMA Network Open.*

eFigure 6. Time Series: Spending for Readmissions (hospital component only), 2008-2015

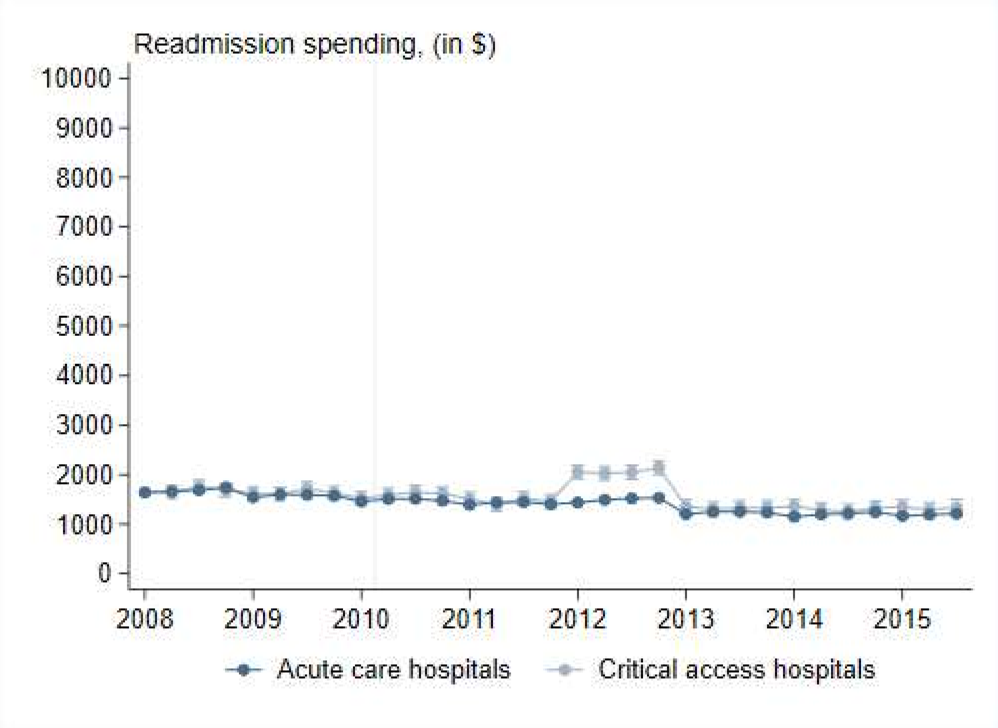

© 2020 Ibrahim AM et al. *JAMA Network Open.*

eFigure 7. Time Series: Spending for Postacute Care, 2008-2015

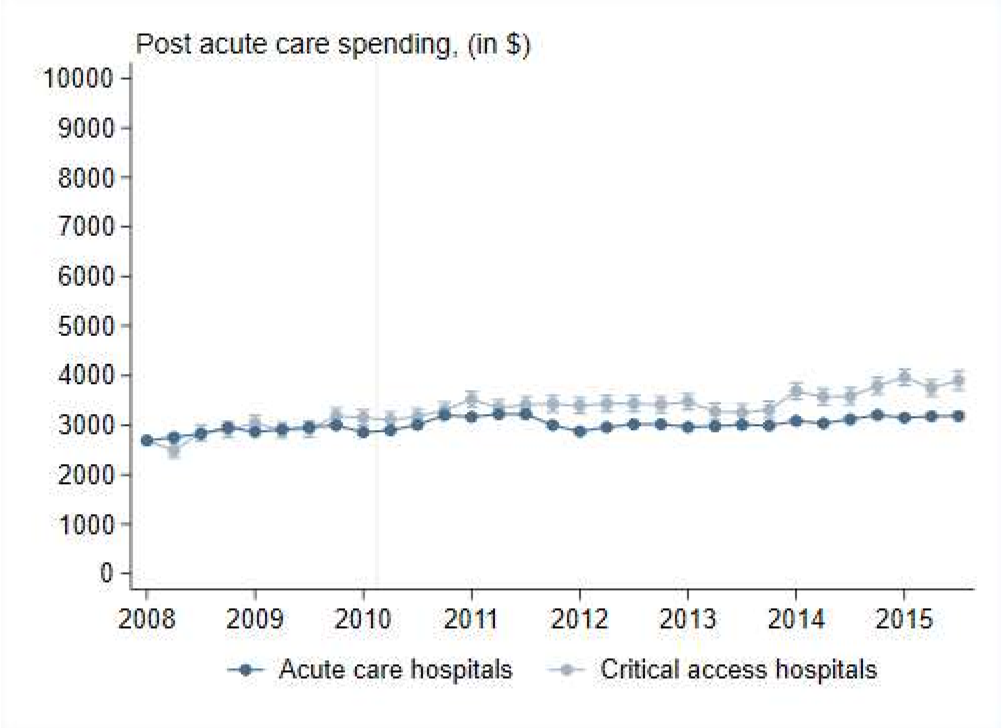

© 2020 Ibrahim AM et al. *JAMA Network Open.*

eFigure 8. Time Series: Spending for Hospital Outpatient Care, 2008-2015

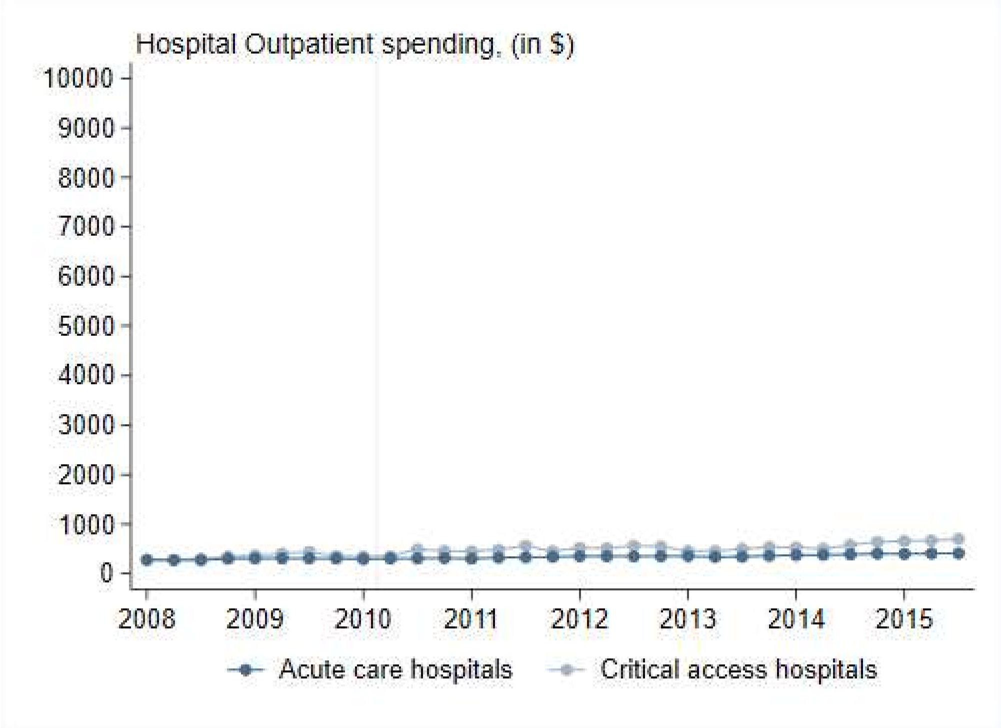

© 2020 Ibrahim AM et al. *JAMA Network Open.*

eFigure 9. Latent Factor Values From Generalized Synthetic Model

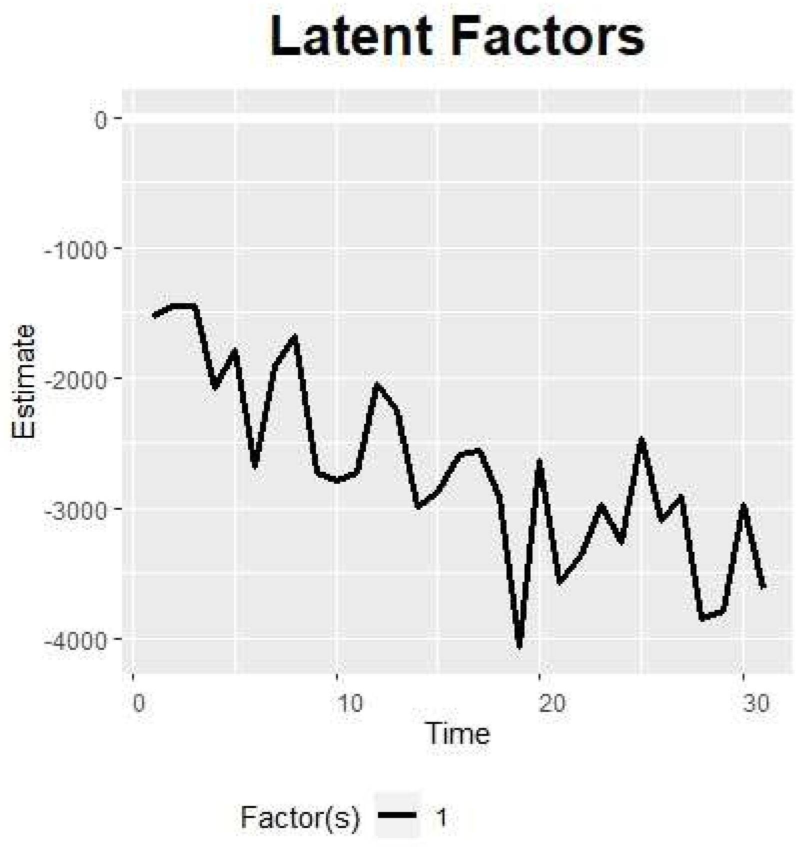

© 2020 Ibrahim AM et al. *JAMA Network Open.*

eFigure 10. Factor Loadings From Generalized Synthetic Control Model

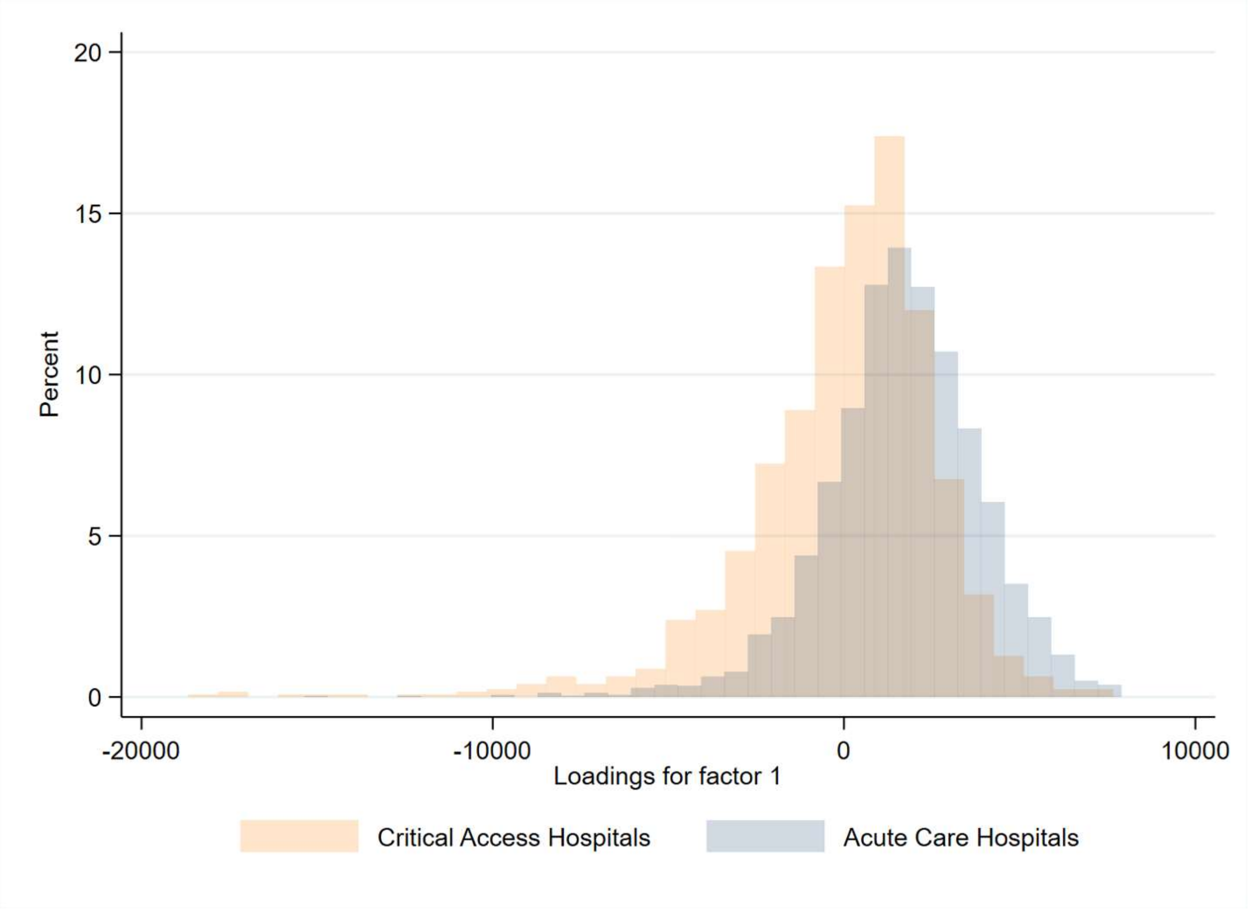

© 2020 Ibrahim AM et al. *JAMA Network Open.*

eFigure 11. Implausible Counterfactual Estimates From Generalized Synthetic Control Model Estimated for Index Admission Spending

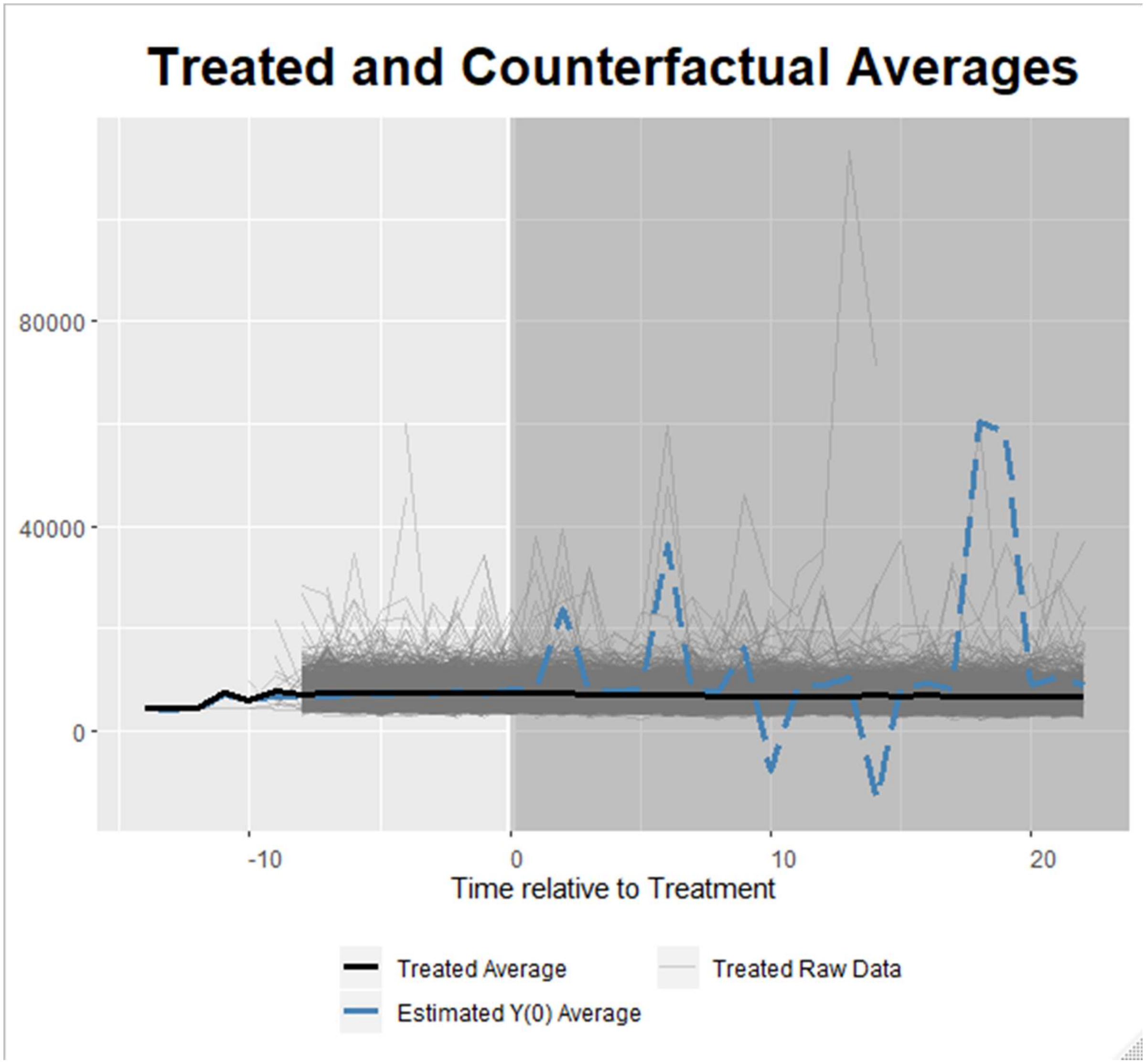

© 2020 Ibrahim AM et al. *JAMA Network Open.*

eTable 1. Parallel Trends Tests Between Targeted and Untargeted Outcomes and Between Acute Care and Critical Access Hospitals

eTable 1A. Targeted and Untargeted Outcomes

Outcome	Targeted conditions trend	Non-targeted conditions trend	Difference in trends, estimate	Difference in trends, lower bound 95% CI	Difference in trends, upper bound 95% CI
Index Hospitalization	-26.36	-21.94	-4.43	-8.43	-0.42
Physician Services, Inpatient	4.58	7.51	-2.93	-4.37	-1.49
Physician Services, Outpatient	0.41	-0.12	0.53	-1.90	2.96
Readmissions	-17.68	-23.62	5.94	-2.33	14.21
Hospital outpatient	3.87	3.65	0.22	-1.27	1.71
Post-Acute Care Services	14.56	31.40	-16.85	-25.64	-8.05
Total 30-Day Episode	-20.64	-3.12	-17.51	-32.37	2.65

© 2020 Ibrahim AM et al. *JAMA Network Open.*

eTable 1B. Acute Care and Critical Access Hospitals

Outcome	Acute care hospital trend	Critical access hospital trend	Difference in trends, estimate	Difference in trends, lower bound 95% CI	Difference in trends, upper bound 95% CI
Index Hospitalization	-24.10	77.01	-101.11	-107.75	-94.47
Physician Services, Inpatient	6.45	5.13	1.32	1.02	3.65
Physician Services, Outpatient	-0.47	-1.22	0.75	-3.16	4.66
Readmissions	-24.83	-12.22	-12.61	-26.40	1.18
Hospital outpatient	3.33	14.00	-10.67	-13.15	-8.20
Post-Acute Care Services	27.64	68.20	-40.56	-55.36	-25.75
Total 30-Day Episode	-11.97	150.91	-162.87	-187.67	-138.08

© 2020 Ibrahim AM et al. *JAMA Network Open.*

eTable 2. DRGs Included in Price Index

DRGs
65
190
191
192
194
195
247
291
292
310
312
313
378
392
470
603
641
683
690
871

© 2020 Ibrahim AM et al. *JAMA Network Open.*

eTable 3. Levels of Preintervention Spending for Statistical Comparisons of Association Between ACA and Hospital Episode Spending

Hospital population and Comparison	Patient population	Cohort	Pre-intervention levels, mean
**Acute care only:** Difference-in-differences, targeted versus untargeted diagnoses	All diagnoses	Comparison group<br>Treatment group	15,013<br>15,007
**Acute care and Critical Access:** Difference-in-differences, acute care versus critical access	All diagnoses	Comparison group<br>Treatment group	12,194<br>15,034
	Targeted diagnoses	Comparison group<br>Treatment group	12,754<br>15,007
	Untargeted diagnoses	Comparison group<br>Treatment group	12,073<br>15,013
**Acute care and Critical Access:** Generalized synthetic control, acute care versus critical access	All diagnoses	Comparison group<br>Treatment group	12,192<br>15,032
	Targeted diagnoses	Comparison group<br>Treatment group	12,772<br>15,015
	Untargeted diagnoses	Comparison group<br>Treatment group	12,056<br>15,011

© 2020 Ibrahim AM et al. *JAMA Network Open.*

eTable 4. Difference-in-Differences Analysis of Changes in Hospital Prices Following Reforms

Outcome	Model specification	DID estimate (95% CI)
Price standardized index hospitalization spending	Model adjusted for patient and hospital characteristics	-695 (-732, -659)
Price standardized index hospitalization spending	Unadjusted	-696 (-773, -659)
Actual (unstandardized) index hospitalization spending	Unadjusted	-930 (-1012, -847)

© 2020 Ibrahim AM et al. *JAMA Network Open.*

eTable 5. Estimated Annual Changes in Spending by Medicare After the ACA by Payment Component

Hospital population and Comparison	Patient population	Estimate	Episodes in the post period to which the estimate applies	Estimated annual savings
**Acute care only:** DID, targeted versus untargeted	All diagnoses	$431	689,554	$ 274,336,407
**Acute care and Critical Access:** DID, acute care versus critical access	All diagnoses	$1,820	5,042,933	$ 8,472,127,440
	Targeted diagnoses	$1,971	689,554	$ 1,245,563,939
	Untargeted diagnoses	$1,949	3,816,613	$ 6,866,380,373
**Acute care and Critical Access:** Generalize synthetic control, acute care versus critical access	All diagnoses	$1,232	4,992,113	$ 5,677,184,507
	Targeted diagnoses	$995	682,242	$ 626,613,037
	Untargeted diagnoses	$1,147	3,778,761	$ 4,000,835,877

To estimate the total annual savings to Medicare, we took our estimate of the effect of the ACA on spending and multiplied it by the number of annual episodes to which the estimate applied in the post-ACA period. Because we used the 20% file, we calculated the number of annual episodes to which an estimate applied by multiplying our study sample by 5.

Medicare Savings = Total Episode Savings x Number of Annual episodes

= Total Episode Savings x (episodes in sample x 5) / years in the post period

Savings for the generalized synthetic estimator for all diagnoses:

= [$1,232 x (4,992,113 x 5)] / (65/12)

= $5,677,184,507 *in annual savings*

© 2020 Ibrahim AM et al. *JAMA Network Open.*

eTable 6. Analysis of CBO Estimates in Billions of Dollars

Type of Spending	Spending Subcategory	Approximate FY 2010-2015 Yearly Average
**Total**	**All Applicable Providers**	**-$12.12**
	**Hospital Providers**	**-$6.68**
**Budget Sequestration Fee Cuts:** Estimate of Total Medicare Spending Cuts, Among Medicare Spending Cuts Subject to 2% Cut Ceiling	All Applicable Providers	-$5.67
	Hospital Providers	-$1.82
**Relevant Non-Health Insurance Coverage ACA Provisions**	Revision of Certain Market Basket Updates and Incorporation of Productivity Improvements into Market Basket Updates That Do Not Already Incorporate Such Improvements (effect of productivity adjustment for home health included in estimate for section 3131) --- *All Applicable Providers* (ACA Fee Cuts; Section 3401 and Section 10319, although latter section not mentioned in analyses)	-$6.50
	Revision of Certain Market Basket Updates and Incorporation of Productivity Improvements into Market Basket Updates That Do Not Already Incorporate Such Improvements --- *Hospital Providers* (ACA Fee Cuts; Section 3401 and Section 10319, although latter section not mentioned in analyses)	-$4.91
	Payment Adjustment for Conditions Acquired in Hospitals (HACRP; Section 3008)	-$0.03
	Medicare Shared Savings Program (MSSP; Section 3022)	-$0.17
	Hospital Readmissions Reduction Program (HRRP; Section 3025)	-$0.25
	Hospital Value-Based Purchasing Program (HVBP Program; Section 3001)	$0.00
	Payment for Qualifying Hospitals (Section 1109)	$0.27
	Payment Adjustment for Low-Volume Hospitals (Section 3125)	$0.05

© 2020 Ibrahim AM et al. *JAMA Network Open.*

		Hospital Wage Index Improvement (Section 3137)	$0.03
		Protections for Frontier States (Section 10324) (This estimate relates to hospitals, hospital outpatient department services, and physicians' services in frontier states)	$0.15

© 2020 Ibrahim AM et al. *JAMA Network Open.*
